# The Role of Gut Microbiota in the Development and Treatment of Obesity and Overweight: A Literature Review

**DOI:** 10.3390/jcm14144933

**Published:** 2025-07-11

**Authors:** Gabriela Augustynowicz, Maria Lasocka, Hubert Paweł Szyller, Marta Dziedziak, Agata Mytych, Joanna Braksator, Tomasz Pytrus

**Affiliations:** 1 Student Scientific Group of Pediatric Gastroenterology and Nutrition, Wroclaw Medical University, 50-369 Wroclaw, Poland; gabriela.augustynowicz@interia.pl (G.A.); marysialasocka@gmail.com (M.L.); marta.dziedziak@student.umw.edu.pl (M.D.); agata.mytych@student.umw.edu.pl (A.M.); 2 2nd Clinical Department of Paediatrics, Gastroenterology and Nutrition, Wroclaw Medical University, 50-369 Wrocalw, Poland; joanna.braksator@umw.edu.pl (J.B.); tomasz.pytrus@umw.edu.pl (T.P.)

**Keywords:** gut microbiota, dysbiosis, obesity, probiotics, fecal microbiota transplantation, FMT

## Abstract

The gut microbiota, dominated by bacteria from the Firmicutes, Bacteroidetes, Proteobacteria, and Actinobacteria phyla, plays an essential role in fermenting indigestible carbohydrates, regulating metabolism, synthesizing vitamins, and maintaining immune functions and intestinal barrier integrity. Dysbiosis is associated with obesity development. Shifts in the ratio of Firmicutes to Bacteroidetes, particularly an increase in Firmicutes, may promote enhanced energy storage, appetite dysregulation, and increased inflammatory processes linked to insulin resistance and other metabolic disorders. The purpose of this literature review is to summarize the current state of knowledge on the relationship between the development and treatment of obesity and overweight and the gut microbiota. Current evidence suggests that probiotics, prebiotics, synbiotics, and fecal microbiota transplantation (FMT) can influence gut microbiota composition and metabolic parameters, including body weight and BMI. The most promising effects are observed with probiotic supplementation, particularly when combined with prebiotics, although efficacy depends on strain type, dose, and duration. Despite encouraging preclinical findings, FMT has shown limited and inconsistent results in human studies. Diet and physical activity are key modulators of the gut microbiota. Fiber, plant proteins, and omega-3 fatty acids support beneficial bacteria, while diets low in fiber and high in saturated fats promote dysbiosis. Aerobic exercise increases microbial diversity and supports growth of favorable bacterial strains. While microbiota changes do not always lead to immediate weight loss, modulating gut microbiota represents an important aspect of obesity prevention and treatment strategies. Further research is necessary to better understand the mechanisms and therapeutic potential of these interventions.

## 1. Introduction

The human microbiota is a complex ecosystem of microorganisms—mainly bacteria, but also archaea, fungi, and viruses that inhabit various areas of the body, including the skin, oral cavity, respiratory tract, urogenital tract, and gastrointestinal tract. The most important is the gut microbiota, which consists of hundreds of types of bacteria, predominantly Firmicutes and Bacteroidetes. It is responsible for key metabolic, immune, and hormonal functions of the host [1,2].

The composition of the microbiota is not constant—it is influenced by factors such as diet, age, lifestyle, disease, and medications (especially antibiotics). Disturbances in its structure and function, referred to as dysbiosis, can contribute to the development of numerous diseases, including obesity [1,2].

Obesity, one of the most serious public health problems, affects hundreds of millions of people worldwide. According to the WHO, more than 340 million children and adolescents aged 5–19 are overweight [3], and in Poland, 62% of men and 46% of women are overweight or obese [4]. Overweight and obesity in adults can be defined by body mass index (BMI), which is at least 25 kg/m^2^ for overweight and at least 30 kg/m^2^ for obesity [3].

A key milestone in research on the link between gut microbiota and obesity was the study by Turnbaugh et al. (2006), which demonstrated that obese mice exhibited an increased Firmicutes to Bacteroidetes (F/B) ratio, and that fecal transplantation from these mice into germ-free mice led to weight gain in the recipients [4]. In subsequent years, large initiatives such as the Human Microbiome Project (HMP) and the European MetaHIT project enabled detailed mapping of the diversity and metabolic functions of the human gut microbiome [5,6]. Numerous studies confirmed associations between microbiota composition and host metabolic status, including reduced microbial diversity and altered abundance of selected taxa in individuals with obesity [7].

The mechanisms linking gut microbiota with obesity are multifaceted. Short-chain fatty acids (SCFAs), such as acetate, propionate, and butyrate, are produced through bacterial fermentation of dietary fiber and influence energy metabolism, appetite regulation, enhancement of intestinal barrier integrity, and exhibit anti-inflammatory effects [8]. Reduced butyrate levels are associated with impaired intestinal epithelial integrity and the chronic inflammation characteristic of obesity [9]. Beyond SCFAs, microbiota-derived metabolites such as secondary bile acids act as signaling molecules regulating glucose and lipid metabolism through FXR and TGR5 receptors [10]. Other bacterial-derived compounds, including trimethylamine N-oxide (TMAO), imidazole propionate, and indole derivatives, have been linked to metabolic dysfunction and insulin resistance [8,11].

Another important mechanism is increased intestinal permeability (“leaky gut”), which allows lipopolysaccharides (LPS)-components of the outer membrane of Gram-negative bacteria to translocate into the bloodstream. LPS activates Toll-like receptor 4 (TLR4) and the NF-κB inflammatory cascade, inducing a chronic low-grade inflammatory state that promotes insulin resistance and fat accumulation [12,13]. Disruption of tight junction protein expression and SCFA deficiency exacerbate barrier dysfunction, aggravating metabolic endotoxemia and complications such as non-alcoholic fatty liver disease (NAFLD) and metabolic syndrome [13,14,15].

However, it is important to note that the hypothesis of a universal increase in the Firmicutes to Bacteroidetes ratio in obesity has been questioned. Cohort studies in humans have yielded inconsistent results, influenced by factors such as diet, age, ethnicity, and geographic location [16]. For example, the Multiethnic Cohort Adiposity Phenotype Study found no predictable shifts in the F/B ratio according to body mass index (BMI), underscoring the complexity and multifactorial nature of microbiota–host interactions in obesity [11].

Meta-analyses highlight the heterogeneity of microbiome alterations in obesity and emphasize the need for personalized approaches in future research and microbiota-targeted therapies [17]. Consequently, although the gut microbiota plays a significant role in the pathogenesis of obesity, the exact microbial patterns and mechanisms remain largely incompletely understood.

In recent years, there has been growing interest in the influence of the gut microbiota on the pathogenesis of obesity. The aim of this paper is to present current data on the mechanisms by which the microbiota can influence metabolism and the potential therapeutic possibilities based on its modulation.

The literature included in the review was searched in databases with a focus on clinical trials and original articles, followed by meta-analyses then literature reviews. The time range of papers recruited for the review assumed a limit of 10 years, of which some articles of special or historical interest were included despite exceeding the limit set. The following keywords were used in the database search: gut microbiota, dysbiosis, obesity, probiotics, fecal microbiota transplantation, FMT. Articles not directly related to the topic, not available in English, and outdated articles were not included. With the above restrictions, the authors selected 100 articles.

## 2. Composition of the Gut Microbiota

The human gut microbiota is an extremely complex ecosystem of microorganisms, dominated by bacteria belonging to several main types. At least 800 different species of bacteria can be found in the intestines of a healthy person [1]. The most numerous group are bacteria of the Firmicutes and Bacteroidetes types, which together account for about 90% of the entire gut microbiota. Firmicutes include bacteria of the Clostridium, *Lactobacillus*, and Ruminococcus genera, while Bacteroidetes are primarily bacteria of the Bacteroides genus [1,2].

In addition to these dominant types, bacteria of the Actinobacteria (e.g., *Bifidobacterium*, *Coriobacterium*) and *Proteobacteria* (e.g., *Escherichia*, *Klebsiella*, *Enterobacter*) types also play an important role. They occur in smaller quantities, but their presence is important for maintaining the microbiological balance of the intestines. It is also worth mentioning the presence of other microorganisms, such as archaea (e.g., *Methanobrevibacter smithii*) and fungi (e.g., *Candida*), which are also part of the gut microbiota, but their role is less well understood [3,18].

The composition of the gut microbiota is dynamic and changes throughout a person’s life. In newborns, aerobic bacteria such as *Enterobacteriaceae dominate*, and as they mature, the microbiota becomes more diverse, with a predominance of anaerobic bacteria such as *Bacteroides* and *Firmicutes*. Around the age of three, the microbiota reaches a composition similar to that of an adult. In older people, there is a decrease in the diversity of the microbiota and a reduction in the number of beneficial bacteria, such as *Bifidobacterium*, which can weaken the immune system and deteriorate health [19].

The composition of the gut microbiota is influenced by many external factors, such as diet, lifestyle, antibiotic use, stress, and environmental exposure. A diet rich in fiber, vegetables, fruits, and fermented products promotes the growth of beneficial bacteria such as *Lactobacillus* and *Bifidobacterium* and increases the production of short-chain fatty acids (SCFAs), which have anti-inflammatory effects. In contrast, a Western diet rich in saturated fats, sugars, and processed foods can lead to dysbiosis, or an imbalance in the microbiota, which is associated with the development of metabolic diseases such as obesity, type 2 diabetes, and cardiovascular disease [20]. The factors that have been described are summarized in Table 1.

## 3. Functions of the Gut Microbiota

Microorganisms colonizing the intestines play a very important role in maintaining homeostasis and the proper functioning of the human body. Intestinal bacteria participate in the processes of digestion and fermentation of dietary fiber, resulting in the production of short-chain fatty acids (SCFA), such as butyrate, propionate, and acetate. These compounds are an important source of energy for intestinal epithelial cells and also have a beneficial effect on lipid and glucose metabolism, which may be important in the prevention of metabolic diseases, including obesity and type 2 diabetes [14].

The gut microbiota is also involved in the synthesis of certain vitamins, especially B vitamins and vitamin K, which are essential for many metabolic processes and the overall functioning of the body [22].

A study by Magnúsdóttir et al. showed that many species of human intestinal bacteria have the ability to synthesize B vitamins such as thiamine (B1), riboflavin (B2), niacin (B3), pantothenic acid (B5), pyridoxine (B6), biotin (B7), folic acid (B9), and cobalamin (B12). Genomic analysis of over 250 strains of gut microbiota revealed that 40 to 65% of them can synthesize at least one of these vitamins, and different strains often complement each other metabolically, jointly contributing to the host’s needs. These results highlight the importance of the gut microbiota as a potential source of vitamins, especially in conditions of limited intake or impaired nutrient absorption [23].

In addition to their metabolic functions, gut bacteria play a fundamental role in shaping and modulating the immune response. They support the development of the immune system and participate in maintaining its proper activity, protecting the body from both pathogens and excessive inflammatory reactions. Commensal bacteria can also occupy ecological niches that are potentially available to pathogens. These bacteria coexist with pathogens in host organisms, forming a complex network of interactions in which they compete for resources and space. In the context of the digestive system, commensal bacteria can occupy spaces that would otherwise be available to pathogens, effectively blocking their colonization. In addition, some commensal bacteria produce antimicrobial substances that directly inhibit the growth of pathogens. However, under certain conditions, such as a weakened host immune system, changes in diet, or antibiotic use, commensal bacteria can become pathogenic. In such situations, previously harmless bacteria can exploit available resources and space, causing infection [24,25].

**Figure 1:**
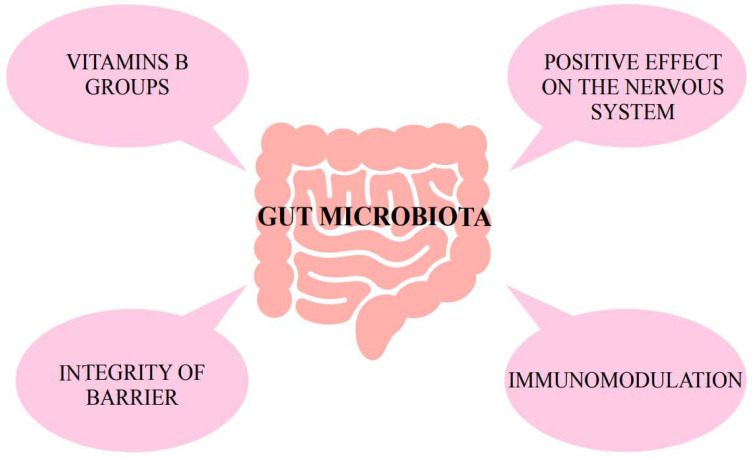
Presentation of the most important functions in whose regulation the microbiota participates. Figure made by the authors based on the literature [14,22,23,24,25,28].

Furthermore, the gut microbiota contributes to maintaining the integrity of the intestinal barrier, limiting the penetration of harmful substances and microorganisms into the bloodstream, thereby reducing the risk of inflammation and infection [26]. Intestinal bacteria are capable of synthesizing neuroactive compounds such as serotonin, dopamine, GABA, and norepinephrine. These neurotransmitters influence the functioning of the gut–brain axis, playing a role in regulating mood, cognitive functions, and behavior [27]. The proper composition and functioning of the gut microbiota are therefore essential for human health—they affect not only metabolism and immunity, but also the body’s protection against pathogens. The most important functions of the microbiota are schematically presented in Figure 1.

### 3.1. Role of Microbiota Metabolites

Experimental studies have shown that short-chain fatty acids (SCFAs), produced by the gut microbiota as a result of dietary fiber fermentation, play an important role in gut–brain communication. In a study by Marcel van de Wouw et al., it was observed that SCFAs, especially butyrate, not only strengthen the integrity of the intestinal barrier, but also influence neurotransmission, microglial activity, and behavior, alleviating stress responses and anxiety symptoms. This indicates the importance of the microbiota in regulating mental and immune functions through neuroimmunological mechanisms [28].

Among the short-chain fatty acids (SCFAs) produced by gut microbiota, butyrate plays a particularly significant role. It serves as the main energy source for colonocytes and supports intestinal barrier integrity by upregulating the expression of tight junction proteins and stimulating mucin secretion [29]. This reduces intestinal permeability and helps prevent the translocation of pathogens and toxins into the bloodstream, thereby lowering the risk of inflammation [30].

Butyrate also exhibits strong anti-inflammatory properties. It inhibits activation of the nuclear factor NF-κB and decreases the production of pro-inflammatory cytokines such as TNF-α and IL-6. Moreover, it promotes the differentiation of regulatory T-cells (Tregs) and modulates immune function through the activation of G-protein-coupled receptors GPR43, GPR109a, and FFAR3 [31].

An increasing number of studies also highlight the role of butyrate in the gut–brain axis. It affects microglial activity and neurotransmission, helping to reduce stress-induced and anxiety-like behavior, thus indicating its relevance in neuroimmune regulation [32].

In metabolic diseases such as obesity, type 2 diabetes, and inflammatory bowel disease (IBD), a reduced production of butyrate and a lower abundance of butyrate-producing bacteria (e.g., *Faecalibacterium prausnitzii*, *Roseburia* spp.) are often observed [28]. Strategies aimed at increasing butyrate levels, such as high-fiber diets, probiotic supplementation, or fecal microbiota transplantation, may have therapeutic potential in these conditions [29]. The role, uses, and properties of SCFAs are summarized in Table 2.

### 3.2. Other Metabolites Produced by the Gut Microbiota

Beyond short-chain fatty acids (SCFAs), the gut microbiota produces a wide array of metabolites that play crucial roles in host physiology. Bile acids, for example, function not only in lipid digestion but also as signaling molecules that act through the FXR and TGR5 receptors to regulate glucose homeostasis, lipid metabolism, and inflammation [33].

Other key microbial metabolites include trimethylamine (TMA), which is converted in the liver into the proatherogenic TMAO; imidazole propionate, which disrupts insulin signaling pathways; and indole derivatives such as indole-3-propionic acid and indole-3-carboxaldehyde, which exert anti-inflammatory and gut barrier-stabilizing effects [34,35]. These metabolites are integral to the gut–host metabolic axis and may serve as biomarkers or therapeutic targets in obesity-related diseases.

### 3.3. The Role of Microbiome Modulation in Inflammatory States

Modulation of the gut microbiota plays a key role in regulating inflammation associated with obesity, metabolic disorders, and cancer. Dysbiosis, or imbalance in the gut microbial community, leads to increased intestinal permeability and the translocation of bacterial toxins—primarily lipopolysaccharide (LPS)—into the bloodstream. LPS activates the immune system via Toll-like receptor 4 (TLR4), inducing a chronic inflammatory response characterized by elevated levels of pro-inflammatory cytokines such as TNF-α, IL-6, and IL-1β [36,37]. In chronic inflammatory conditions like non-alcoholic fatty liver disease (NAFLD), alterations in the gut microbiota are associated with increased hepatic inflammation and the progression of fibrosis. Patients with advanced NAFLD show reduced microbial diversity and decreased abundance of beneficial bacteria such as *Lactobacillus* and *Bifidobacterium*, alongside increased levels of *Ruminococcus* and *Escherichia* species [38]. In metabolic syndrome, dysbiosis contributes to insulin resistance and metabolic disturbances, while targeting the gut microbiome—for example through dietary interventions—offers a promising strategy to reduce inflammation and improve metabolic outcomes [36]. Chronic inflammation also promotes carcinogenesis through several mechanisms, including oxidative DNA damage via reactive oxygen species (ROS), the creation of a tumor-promoting microenvironment through enhanced proliferation and angiogenesis, and modulation of immune responses by influencing the activity of T-cells, macrophages, and dendritic cells. In colorectal cancer (CRC), the presence of specific bacteria such as Fusobacterium nucleatum has been linked to increased local inflammation, more aggressive tumor behavior, and poorer therapeutic response. Animal studies have shown that fecal microbiota transplantation (FMT) from tumor-bearing mice promotes tumor growth in recipients, whereas FMT from healthy donors restores microbial balance and reduces inflammation, offering protection against tumorigenesis [39]. In pediatric leukemia—especially during chemotherapy and hematopoietic stem cell transplantation (HSCT)—gut dysbiosis compromises intestinal barrier integrity, increases LPS translocation, and contributes to persistent inflammation. These changes are associated with a higher risk and severity of infections and graft-versus-host disease (GvHD), as well as reduced clinical outcomes. The loss of beneficial bacteria, such as *Bacteroides*, *Ruminococcaceae*, and butyrate producers, correlates with elevated inflammatory markers, and depletion of key short-chain fatty acids (SCFAs), particularly butyrate and propionate, further worsens inflammation and impairs gut barrier function [37]. Selected diseases, their impact on the composition of the intestinal microbiota and their influence on the clinical condition and inflammatory process are summarized in Table 3.

## 4. Intestinal Dysbiosis and Obesity

Intestinal dysbiosis, or an imbalance in the microbiota, is characterized by changes in the composition and function of intestinal microorganisms. In obese individuals, a reduction in microbiota diversity and changes in the proportions of dominant bacteria, such as an increase in the Firmicutes to Bacteroidetes ratio, are often observed. Such changes can lead to metabolic disorders, inflammation, and an increased risk of developing chronic diseases such as type 2 diabetes and cardiovascular disease [4].

In Riduara’s study, experiments on mice showed that transplanting microbiota from obese individuals to bacteria-free animals led to an increase in body weight in the recipients, even when they were fed the same diet. This suggests that the microbiota may influence the efficiency of energy absorption from food and increase the tendency to become obese [40].

Research on the link between gut microbiota and obesity has been conducted for a long time; a 2005 study by Fredrik Bäckhed, Ruth E. Ley, and Jeffrey I. Gordon examined the effect of obesity on the composition of the gut microbiota. The researchers found that obese individuals have an increased ratio of Firmicutes to Bacteroidetes bacteria, which may affect the efficiency of energy extraction from food. These changes suggest that the gut microbiota plays an important role in the development of obesity by influencing the host’s energy metabolism [41].

One important aspect of the pathogenesis of obesity is reduced gut microbiota diversity. Many studies indicate that obese individuals have poorer and less diverse microflora, which may affect the gut–brain axis, inflammatory responses, and energy metabolism [42]. However, not all analyses confirm a strong correlation between reduced microbiota diversity and the development of metabolic diseases—significant associations are observed in only about one-third of cases [41]. It is worth noting that the intestinal bacterial ecosystem is highly stable and resistant to external factors. Therefore, obesity may be the result of complex functional disorders of the microbiome rather than simply changes in the abundance of specific bacterial strains. Particular importance is attached to *Akkermansia muciniphila*, a bacterium residing in the intestinal mucus layer, which is increasingly seen as a biomarker of metabolic health. Numerous studies indicate that the presence of *A. muciniphila* in the gastrointestinal tract correlates with better metabolic parameters, such as improved insulin sensitivity and a favorable lipid profile [43]. Supplementation with this bacterium in overweight and obese individuals has been associated with improved metabolic indicators, making it a potential candidate for a new generation of probiotics [44,45]. *A. muciniphila* also plays an important role in maintaining the integrity of the intestinal barrier and modulating the immune response, acting as a “guardian” of the intestinal mucosa and preventing the penetration of antigens and toxins into the bloodstream [45].

## 5. Mechanisms of Microbiota Influence on Energy Homeostasis

The gut microbiota plays an important role in regulating the host’s energy metabolism, influencing nutrient absorption, hormone balance, immune responses, and gut–brain axis function. Gut bacteria also influence the secretion of hormones that regulate appetite and metabolism. SCFAs can stimulate the secretion of glucagon-like peptide 1 (GLP-1), which increases satiety and improves insulin sensitivity. The microbiota also participates in the modulation of ghrelin and leptin levels, hormones responsible for hunger and weight control. An important aspect of the microbiota’s action is its influence on the immune system. In the case of dysbiosis, the integrity of the intestinal barrier is disrupted, allowing lipopolysaccharides (LPS) to enter the systemic circulation. The presence of LPS induces chronic low-grade inflammation, which is associated with the development of insulin resistance and obesity [46].

Another mechanism through which the microbiota can influence energy homeostasis is the gut–brain axis, a complex system of communication between the gastrointestinal tract and the central nervous system. It involves neural, hormonal, and immunological mechanisms. The composition of the microbiota modulates SCFA production and influences the activity of intestinal neurons and the secretion of appetite-regulating hormones. Disruptions in this communication, often resulting from dysbiosis, are associated with the pathogenesis of obesity and other metabolic diseases [46,47].

## 6. Differences in Microbiota Composition Between Obese and Lean Individuals

The gut microbiota in healthy individuals is generally highly diverse, whereas a relative lack of diversity in the gut microbiota predisposes to diseases such as obesity [48]. Obese individuals exhibit an altered composition of the gut microbiota compared to lean individuals [44].

In humans, sequencing of microbial ribosomal RNA and DNA has shown that Bacteroidetes and Firmicutes are the two most dominant bacterial phyla in most individuals, accounting for over 90% of the entire community. *Methanobrevibacter smithii* is the most dominant archaeon. Other phyla include Proteobacteria, Actinobacteria, Fusobacteria, and *Verrucomicrobia* [2,49,50]. Among Gram-negative Bacteroidetes are genera such as Bacteroides, Prevotella, Parabacteroides, and Alistipes, whereas Gram-positive Firmicutes include species like *Faecalibacterium prausnitzii*, *Eubacterium rectale*, and *Eubacterium hallii*, among many other low-abundance species [50].

Many studies suggest that an increased Firmicutes/Bacteroidetes ratio is a hallmark of the gut microbiota in obesity, which may influence the efficiency of energy extraction from the diet and its storage as adipose tissue, thereby promoting weight gain [26,44,48,51].

In mouse models, transplantation of gut microbiota from conventionally raised mice into germ-free mice increased fat accumulation and insulin resistance despite limited food intake, confirming the role of microbiota in fat deposition [48]. Moreover, 16S rRNA sequencing revealed that obesity is associated with an increase in Firmicutes and a decrease in Bacteroidetes abundance. Obese mice showed a 50% reduction in Bacteroidetes and a proportional increase in Firmicutes [48,49,52,53]. Similar phenomena have been observed in humans; a higher Firmicutes to Bacteroidetes ratio has been reported in obese children compared to normal-weight peers, in women with overweight/obesity and metabolic syndrome versus those without metabolic syndrome, and in overweight Japanese individuals compared to non-overweight controls [48,50].

Elevated abundances of *Staphylococcus* spp. and *Lactobacillus reuteri* have been reported in obese individuals and positively correlated with energy intake and plasma C-reactive protein (CRP) levels, respectively [49]. Notably, obese subjects exhibited significant decreases in genera such as *Akkermansia*, *Faecalibacterium*, *Oscillibacter*, and *Alistipes* compared to normal-weight individuals [2]. Higher levels of *Lactobacillus* were found in obese patients than in controls; some species within this genus (e.g., *L. reuteri*) were indeed associated with obesity, while others (*L. casei*, *L. plantarum*) correlated with weight loss in humans and animals [2,53].

Million et al. reported that *M. smithii* and *B. animalis* were associated with normal weight, whereas *L. reuteri* was linked to obesity [2,48]. However, other studies have yielded inconsistent results. Some found no significant changes or even a decreased Firmicutes to Bacteroidetes ratio in obese individuals and animals. Many analyses observed reduced gut microbiota diversity in obese patients compared to those with normal weight [49,53]. This suggests that alterations in microbiota composition at the family, genus, or species level may be more relevant to obesity pathogenesis than the Firmicutes/Bacteroidetes ratio alone [49].

Data indicate that the relative abundance of Firmicutes in healthy individuals varies from 11% to 95%, and *Bacteroidetes* from 0.6% to 86.6%. Such high variability complicates the clear assessment of differences in obese individuals. A meta-analysis by Finucane et al. demonstrated that discrepancies among studies exceeded differences between lean and obese subjects within single studies [49].

For example, Zhang et al. found no significant differences in Bacteroidetes abundance between obese and lean individuals, and analysis of data from a US cohort of over 2500 subjects indicated a lower *Firmicutes*/*Bacteroidetes* ratio in obese participants [43]. Koutoukidis et al. reported decreased microbial diversity in obese versus lean individuals but no changes in the Firmicutes to Bacteroidetes ratio [54]. Similarly, Ridaura et al. observed no association between BMI or weight loss and relative populations of major colonic bacterial groups, including Bacteroidetes, in stool samples from obese and non-obese individuals [40].

Significant associations have also been observed between specific microbial genera and obesity or leanness, with population-dependent differences. For instance, genera such as *Faecalibacterium*, *Akkermansia*, and *Alistipes* correlate with lean phenotypes, whereas *Prevotella* and *Ruminococcus* show associations with obesity in Western populations but with leanness in Eastern populations. Interestingly, *Roseburia* and *Bifidobacterium* are linked to leanness in Eastern populations, while *Lactobacillus* has been associated with obesity in Western populations [55,56]. The indicated differences are summarized in Table 4.

It should be noted that the Firmicutes/Bacteroidetes ratio is most often analyzed in terms of predisposition to obesity. However, the relationship between these variables is ambiguous. Despite demonstrating a change in the F/B ratio in obese individuals in both animal and human-based models, no specific cause-and-effect relationship has been demonstrated [41]. The F/B ratio may show high variability between individuals due to differences in research methodology, the influence of diet, medication, and the age of participants [49]. In human-based models, the F/B ratio in obese individuals compared to normal-weight individuals may be lower [48], higher [48,50], or unrelated [52]. Furthermore, the F/B ratio may not reflect actual metabolic and functional changes in the microbiota, which would require confirmation in much more in-depth and precise analyses based on bacterial metabolites, signaling pathways, and enzymatic functions [48,50]. Therefore, the use of the F/B ratio is currently very limited and has not been confirmed in population studies [53].

## 7. Differences in Microbiota Composition Depending on Geographical Region

The gut microbiota varies geographically mainly due to lifestyle, diet, genetics, and climate [49]. Eating habits are considered one of the main factors contributing to the diversity of the human gut microbiota [1]. Since the composition of the gut microbiota of healthy individuals varies depending on the geographical region, it can be assumed that microbial imbalances (dysbiosis) are also specific to a given area [3].

A study of 1000 Israelis with similar eating habits and lifestyles showed that genetic origin has no significant effect on the composition of the gut microbiota. Its overall heritability may be less than 2%, while more than 20% of microbiome variability is associated with diet, medication use, and anthropometric parameters [48].

In the Inuit, a diet rich in protein and fat shapes the specific composition of the microbiota. Meta-analyses indicate that the *Firmicutes*/*Bacteroidetes* ratio is higher at high latitudes, which may reflect an increased ability to obtain energy. Similar changes are observed in animal-based models in mice living in cold conditions or fed a high-fat diet [49].

A study by De Filippo et al. using 16S rDNA sequencing and biochemical analyses showed significant differences in the composition of the gut microbiota of children raised in Africa (Burkina Faso) and Europe (Florence, Italy). The microbiota of children from rural Africa (BF) was adapted to a fiber-rich diet, with a predominance of *Bacteroidetes* and a lower proportion of *Firmicutes* (*p* < 0.001), while the opposite proportions were observed in children from Europe (EU). Children from BF also had higher concentrations of short-chain fatty acids (SCFA) [57].

Analysis of the gut microbiota of lean, overweight, and obese individuals from Argentina, the US, and the UK using 16S rRNA sequencing and bioinformatics tools showed that the composition of the microbiota differs significantly between regions, regardless of BMI. For example, British individuals had lower abundance of bacteria from the Coriobacteriaceae family than Americans in all weight groups. *Actinomycetaceae* was more common in individuals from the United States than in Argentineans and British individuals, and *Streptococcaceae* had higher abundance in the Argentinean population and lower abundance in the British population [58].

Medina D. et al. compared the diversity of the gut microbiome of thin and obese individuals from Chile with data from studies in other regions such as the United States, France, and Saudi Arabia. Data from 16S rRNA sequencing was used. The results of the study show that differences between the gut microbiota of thin and obese people depend on the geographical region. In Chile and France, significant differences were found between BMI groups, with an increase in *Megasphaera* and *Veillonella* bacteria observed in obese people. The changes also affected *Adlercreutzia* and *Lachnospira* (directional differences depending on the country). In contrast, in the US and Saudi Arabia, no significant differences in microbiota composition were found between BMI groups. In addition, the microbiota of obese individuals from each country formed distinct clusters, indicating a clear influence of the local environment [56].

A review of studies conducted by Xu Z et al. shows that the relationship between gut microbiota and obesity differs between the East and the West. The gut microbiota was assessed using 16s rRNA amplicon sequencing or metagenomic shotgun sequencing. The *Coriobacteriaceae* family was associated with an increase in abundance in obese individuals only in the West, while no changes were observed in the East. The *Ruminococcaceae* family was associated with a decrease in abundance in obese individuals (i.e., higher in lean individuals) in the East, while in the West, the results were contradictory—some studies showed an increase, others a decrease. Similarly, the genus *Prevotella* showed a decrease in abundance in obese individuals in the East and an increase in the West. The genera *Ruminococcus* and *Roseburia* were less abundant in obese individuals in the East, but more abundant in obese individuals in the West. In contrast, the *Lactobacillus* genus was generally more abundant in obese individuals in the West, while in the East the results were mixed (both increase and decrease) [59].

## 8. Gut Microbiota Influence on Intestinal Hormones

The gut microbiota plays a crucial role in regulating gastrointestinal functions, including the modulation of intestinal hormone secretion, which affects digestion, appetite, and energy metabolism. Ghrelin, known as the “hunger hormone,” is primarily produced in the stomach and stimulates appetite. Studies suggest that the composition of the gut microbiota can influence ghrelin levels, thereby affecting hunger sensation and food intake [27].

Glucagon-like peptide-1 (GLP-1) is secreted by L-cells in the small intestine in response to nutrient presence and acts as an appetite suppressant as well as a stimulator of insulin secretion. Certain gut bacteria ferment dietary fiber to produce short-chain fatty acids (SCFAs), which may enhance GLP-1 secretion, thus contributing to glycemic control and satiety [27].

Peptide YY (PYY), secreted by L-cells in both the small and large intestines, functions as an appetite suppressant. The presence of specific gut bacteria can modulate PYY levels, playing an important role in the regulation of food intake and body weight control [14].

Cholecystokinin (CCK) is a hormone released by I-cells of the small intestine in response to fats and proteins, stimulating the secretion of pancreatic enzymes and bile, while inhibiting gastric emptying. The gut microbiota may influence CCK secretion, thereby modulating digestive processes and feelings of satiety [14].

Gut dysbiosis can lead to abnormal secretion of intestinal hormones, which has been linked to the development of obesity, type 2 diabetes, and other metabolic disorders. Interventions aimed at modulating the microbiota—such as probiotics, prebiotics, or dietary modifications—may influence intestinal hormone secretion, offering potential therapeutic strategies for these conditions [60].

## 9. Effects of Probiotics and Prebiotics on Microbiota Composition

Probiotics, prebiotics, synbiotics, and postbiotics (PPSP) are powerful regulators of the gut microbiota, giving them potential for preventing metabolic diseases [61].

Elie Metchnikoff suggested that health could be improved by manipulating the gut microbiome with good bacteria found in yoghurt. This became the concept of probiotics in medicine [62]. The current definition of probiotics is ‘live strains of strictly selected microorganisms which, when administered in adequate amounts, confer a health benefit on the host’. Lilly and Stillwell described probiotics as microorganisms that stimulate the growth of other microorganisms [63].

Bacteria such as *Lactobacillus*, *Bifidobacterium* and *Enterococcus* are beneficial and help improve the stability of the gut microbiota [64]. Human probiotic microorganisms mainly belong to the following genera: *Lactobacillus*, *Bifidobacterium*, *Lactococcus*, *Streptococcus*, and *Enterococcus*. In addition, Gram-positive bacterial strains belonging to the genus Bacillus and some yeast strains belonging to the genus Saccharomyces are commonly used in probiotic products [65].

Probiotics are known to stimulate the native intestinal microbiota, strengthen the host’s immunity and help reduce cholesterol [64]. Clinical studies have demonstrated the effectiveness of probiotics in the treatment of diseases such as obesity, insulin resistance syndrome, type 2 diabetes, and non-alcoholic fatty liver disease [65].

There is evidence that consuming dairy products containing probiotics lowers blood cholesterol levels, which may be helpful in preventing obesity, diabetes, cardiovascular disease and stroke. The reduction in cholesterol levels achieved with probiotics is less pronounced than with pharmaceuticals, but it leads to a significant minimization of side-effects [65].

Mann and Spoerig revealed that people who consumed *Lactobacillus* spp. fermented yoghurts had low blood cholesterol levels. Subsequently, Harrison et al. observed a reduction in serum cholesterol in infants consuming *Lactobacillus acidophilus* [64].

Prebiotics do not contain microorganisms, only substances that stimulate their growth [66].

They are non-living food ingredients associated with beneficial modulation of the gut microbiota by selectively stimulating the growth and/or activity of one or more bacteria in the colon to improve the health of the host [64,65,67].

Prebiotics are widely found in several plants, such as onions, asparagus, garlic, chicory, artichokes, oats and wheat [64]. The most popular prebiotics are oligosaccharides found in plants. Indigestible carbohydrates, including polysaccharides (resistant starch, pectin and dextrin) and oligosaccharides such as fructooligosaccharides, galactooligosaccharides, xylooligosaccharides, isomaltooligosaccharides, mannanooligosaccharides, raffinose oligosaccharides, arabinoxylan oligosaccharides, lactulose and inulin, have prebiotic properties [66].

High potential is attributed to the simultaneous use of probiotics and prebiotics.

In 1995, Gibson and Roberfroid introduced the term ‘synbiotic’ to describe the combination of synergistically acting probiotics and prebiotics. A selected ingredient introduced into the digestive tract should selectively stimulate the growth and/or activate the metabolism of the physiological intestinal microbiota, thus exerting a beneficial effect on the health of the host [65].

Parnell and Reiner demonstrated that prebiotic supplementation reduced total cholesterol levels in hypercholesterolemic rats. Diets containing 0, 10, or 20% prebiotic fiber reduced serum cholesterol levels by approximately 25%, and obese rats with 10% supplementation also showed a 40% reduction in liver fat. These effects were associated with increased intestinal content and regulation of genes responsible for cholesterol and bile metabolism. Obesity is often associated with the progression of cardiovascular disease, and both probiotic and prebiotic intake had anti-obesogenic effects [66].

Studies analyzed by John GK et al. indicate that the use of probiotics, prebiotics or synbiotics is associated with a statistically significant reduction in BMI and body weight compared to placebo. The strongest effects were observed with probiotic supplementation (a decrease in BMI of 0.33 and body weight of 0.65 kg). Prebiotics showed a weaker but still significant effect on weight reduction, although their effect on BMI was marginal. Probiotic and synbiotic supplementation may support weight and BMI reduction, complementing traditional methods of obesity treatment [67].

The study by Jagielski et al. involved 56 obese women (aged 25–45), divided into two groups. Group A (*n* = 31) followed a low-calorie diet (1100–1300 kcal) enriched with probiotic and prebiotic products (fermented milk drinks, pickled vegetables, groats, lentils, fish oil, highly mineralized water). Group B (*n* = 25) followed a standard weight-loss diet. After 3 months, both groups reported beneficial changes in their eating habits and similar reductions in body weight, body fat, and waist circumference. The key factors for these effects were primarily: a reduction in the calorie content of the diet, regular meals, and a higher consumption of vegetables, fruit and whole grains—and not necessarily the presence of fermented products alone [68]. The differences between the effects of the different drug groups on the microbiota is summarized in Table 5

## 10. Incretin-Based Medicaments

Incretin-based drugs, including GLP-1 receptor agonists and dual GLP-1/GIP agonists, play an important role not only in glycemic control and body weight management but also in modulating the gut microbiota. Studies suggest that the metabolic effects of these drugs may be partially mediated through their influence on the composition and function of the gut microbiome.

Liraglutide increases the abundance of bacteria with potentially beneficial effects, such as *Bacteroides*, *Ruminococcus*, and *Akkermansia muciniphila*, which is associated with improvements in metabolic parameters and reduced inflammation [69]. A similar effect has been observed for semaglutide, which increases levels of *A. muciniphila* while simultaneously reducing microbiota diversity, potentially impacting gut barrier function [70]. Exenatide promotes the growth of bacteria linked to better metabolism, although some studies also report an increase in pro-inflammatory strains.

Tirzepatide, a dual agonist of both GLP-1 and GIP receptors, shows particularly promising effects—in animal models, it improved gut microbiota composition and bile acid metabolism, contributing to reductions in liver steatosis and improvements in insulin resistance [71].

The mechanisms of action of incretin-based drugs include increased production of short-chain fatty acids (SCFAs) such as butyrate, propionate, and acetate; enhancement of intestinal barrier integrity via upregulation of tight junction proteins and colonization by *A. muciniphila*; and anti-inflammatory effects through the reduction in pro-inflammatory cytokines (TNF-α, IL-6) [12,13,70]. The impact of these drugs on the gut microbiota may represent an additional component of their therapeutic efficacy in the treatment of obesity and type 2 diabetes.

## 11. Fecal Microbiota Transplantation

FMT (Fecal Microbiota Transfer) can be used as a treatment option for obesity and other metabolic diseases [44]. FMT aims to restore microbial balance by transferring healthy and diverse gut microbiota from a donor to a recipient [52]. FMT is performed from lean donors to overweight individuals [44] because the ‘lean microbiome’ has been associated with reduced adipose tissue, while the ‘obese microbiome’ is associated with increased fat storage and metabolic dysfunction [55]. Importantly, careful selection of FMT donors ensures the safety of the treatment process [44].

Modulation of the gut microbiota may improve glycaemic control and promote satiety by increasing SCFA production, influencing GLP-1, altering bile acid composition, and reducing inflammation in adipose tissue [44,72].

Experiments with faecal microbiota transplantation (FMT) in rodents confirm the involvement of the gut microbiota in weight loss. Transferring faeces from mice or humans after bariatric surgery to germ-free mice resulted in weight loss in the recipients, although less than after surgery alone. This demonstrates the important role of the gut microbiota in weight regulation [44].

FMT in obese mice improves metabolic parameters, reduces fat mass, and increases insulin sensitivity, while simultaneously changing the composition of the gut microbiota. In humans, FMT in capsule form from a lean donor was found to be safe and well tolerated, affecting the bile acid profile and microbiota, but did not significantly change BMI after 12 weeks [55]. In a placebo-controlled study by Allegretti JR et al., FMT in capsule form was administered to obese individuals (the material came from lean individuals). Significant changes in the gut microbiome and bile acid profile were demonstrated, which were similar to those in the lean donor. However, no significant changes in body weight, insulin sensitivity or other metabolic indicators were observed during the observation period. This means that FMT alone did not cause weight loss in the short term [72].

Similar conclusions were also drawn from a study conducted by Yu EW, et al. [51].

The study by Rinott E et al. examined the effect of autologous FMT (aFMT) on weight maintenance after weight loss during a 6-month diet period. The diet alone increased the diversity of gut bacteria, increased the number of bacteria producing short-chain fatty acids, and reduced the Firmicutes/Bacteroidetes ratio, which in some previous studies has been associated with obesity. Autologous FMT after prior weight loss consolidated the beneficial changes in the composition of the gut microbiota, especially those related to the diversity and presence of metabolism-supporting bacteria. However, these changes were not sufficient to completely stop the yo-yo effect, although they may delay or mitigate weight regain [73].

In a study by Zuppi et al., FMT from lean, healthy donors was used in obese individuals. The aim of the study was not only to assess the effect of FMT on the bacterial composition of the microbiome, but also to analyze changes in the bacteriophage population, which are less frequently considered in such interventions. Microbiota transplantation caused significant changes in bacteriophage populations, which proved to be more lasting and pronounced than changes in the composition of intestinal bacteria. Despite the observed changes in the structure of the microbiome, no clear metabolic benefits or weight loss were reported in the study participants. The results suggest that although FMT affects the gut microbiome, especially its viral component, its modification alone was not sufficient to achieve rapid clinical effects in the treatment of obesity [74].

Fecal microbiota transplantation (FMT) is an effective and approved treatment for recurrent *Clostridioides difficile* infections [75]. However, its use in other dysbiosis-related conditions, such as metabolic syndrome or inflammatory bowel disease, faces numerous limitations [76].

First of all, FMT, although more comprehensive than probiotics, is associated with technical and logistical challenges. Traditional methods of administration (colonoscopy, enemas, probes) are invasive and burdensome for both patients and medical staff. Oral capsules, although more convenient, are characterized by limited stability (especially frozen forms) and the need for long-term use of lyophilisates [76].

Clinical trials to date, apart from C. difficile, are characterized by small sample sizes, lack of dietary standardization, and short follow-up times, which translates into ambiguous and short-lived clinical effects [76]. In addition, the effectiveness of donor strain colonization is limited in patients with high baseline microbiota diversity or after previous dietary interventions that may stabilize the microbiome and inhibit the implantation of new strains [77].

Furthermore, a single FMT procedure appears to be insufficient for the permanent treatment or prevention of diseases; therefore, dietary interventions may be a necessary complement to therapy, supporting the effectiveness and durability of the effects [78]. The mechanisms of microbiome modulation after FMT remain poorly understood, and the methods used, such as 16S rRNA sequencing, do not provide sufficient taxonomic resolution to accurately track the colonization of individual donor strains [75].

In addition, there are significant ethical and regulatory barriers regarding informed consent, data protection, and the lack of official recognition of FMT as a therapeutic standard, which limits its wider use. Raising awareness among physicians and patients is crucial for the proper assessment of the risks and benefits of this method [76].

In summary, FMT is a promising therapeutic strategy, but its effectiveness depends on the route of administration, the host’s microbiota profile, dietary interventions, the number of treatments, and precise microbiological control. Further research is needed to optimize therapeutic protocols and identify patients who may benefit most from this form of treatment [76,77,79].

## 12. Dietary Treatment

**Figure 2:**
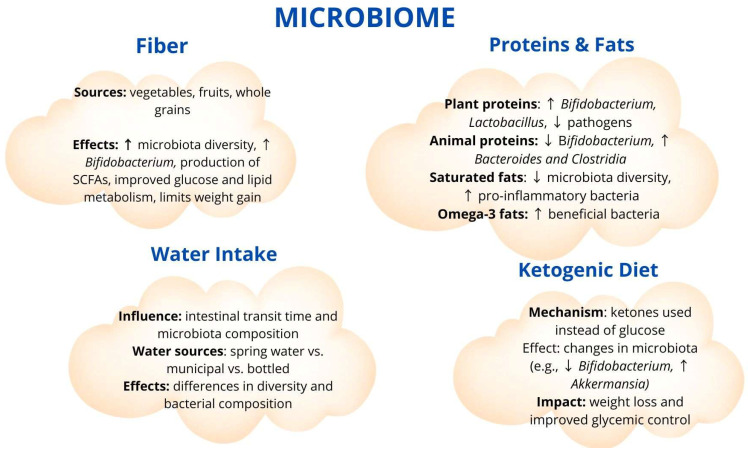
Influence of selected dietary components on the qualitative composition of the human gut microbiota.

Diet is one of the main modulators of the composition of the gut microbiota, which directly affects the host’s homeostasis and biological processes, but also through metabolites derived from the microbial fermentation of nutrients—in particular, short-chain fatty acids (SCFAs) [80]. A summary of the impact of selected nutrients is summarized in Figure 2.

Prebiotics, probiotics, and synbiotics are not the only compounds that affect the gut microbiota. Changes also occur in connection with the consumption of adequate amounts of dietary fiber, diverse sources of protein, and unsaturated fats, with particular emphasis on omega-3 (ω-3) fatty acids [81].

### 12.1. Fiber

Fiber generally refers to indigestible complex carbohydrates from plants [82].

Different types of fiber and the short-chain fatty acids (SCFA) produced by their fermentation have a beneficial effect on the host’s metabolism, supporting glucose and lipid regulation [83]. Diets rich in fiber are generally associated with increased microbiota diversity in humans [82]. Diet can also have a negative impact on health [82]. One example is the Western diet, in which the increase in calorie-dense and ultra-processed foods rich in saturated fats, high in salt, rich in processed carbohydrates, and low in fiber is correlated with the rapidly increasing prevalence of obesity, type II diabetes, metabolic syndrome, and CVD in industrialized countries [82,83]. These effects are likely caused by both factors independent of the gut microbiota and factors dependent on it [82]. Excessive consumption of carbohydrates in the Western diet, rich in refined grains, starch and added sugar, has a negative impact on the gut microbiota [84]. Dietary modification, including increased fiber intake, is an effective strategy for modulating the microbiota to improve health [83]. There is a positive association between total dietary fiber per kilocalorie of energy and an increase in *Bifidobacterium* abundance [81]. A diet containing indigestible carbohydrates, rich in whole grains and bran, is associated with an increase in Bifidobacteria and Lactobacilli in the gut [85]. Interestingly, a study of healthy individuals found that weight gain was inversely correlated with dietary fiber intake in the long term, proving that fiber plays a role in limiting weight gain in the long term [83].

### 12.2. Proteins and Fats

The fermentation of animal proteins reduces the number of *Bifidobacterium* and SCFA production. Plant proteins, such as those from peas, promote the growth of *Bifidobacterium* and *Lactobacillus*, reduce pathogenic bacteria such as Bacteroides fragilis and Clostridium perfringens, and support SCFA production [19,80]. A lower number of *Bifidobacterium* adolescentis and an increased number of Bacteroides and Clostridia have been shown in people consuming a diet rich in beef compared to those consuming a meat-free diet [19,80,85]. Most studies have shown that protein intake correlates positively with overall microbial diversity. For example, it has been reported that consumption of whey and pea protein extract increases the number of commensal *Bifidobacterium* and *Lactobacillus* bacteria in the gut, while whey additionally reduces the number of pathogenic Bacteroides fragilis and Clostridium perfringens bacteria [85]. The typical Western diet is rich in saturated and trans fats and low in monounsaturated and polyunsaturated fats, which predisposes consumers to many health problems [85]. Obesity-related dysbiosis is directly linked to a high-fat diet (HFD) and manifests itself in a reduced overall number of microbiota, which is a change in the abundance of bacterial species [86]. Changes in the gut microbiota are often assessed based on the Firmicutes/Bacteroidetes ratio, which increases in the case of a high-fat diet (HFD), which may indicate microbial imbalance [86]. As indicated by Rinninella et al. (2019), the effect of fat on the microbiota depends on its type: saturated fats reduce its diversity and promote the proliferation of pro-inflammatory bacteria, such as *Bilophila wadsworthia*, while unsaturated fats, especially omega-3 fatty acids, support the growth of beneficial bacteria, including *Bifidobacterium*, and have anti-inflammatory effects [19,80]. In obesity studies traditionally comparing low-fat diets with high-fat diets, a study in rats showed that a low-fat/high-sucrose diet led to a reduction in bacterial diversity, an increase in the Firmicutes: Bacteroides, a proliferation of Ruminococcaceae, intestinal inflammation, altered gut–brain communication and obesity, similar to an isocaloric high-fat/high-sucrose diet [84]. The ketogenic diet (KD), by limiting carbohydrate intake to 5–10% of daily calorie intake, leads to a state of ketosis, in which the body uses ketone bodies instead of glucose as its main source of energy [86,87,88]. This mechanism promotes weight loss, improves glycaemic control, and may affect the composition of the gut microbiota [88]. The results of studies on the effect of the ketogenic diet on the gut microbiota are inconclusive—although significant changes have been demonstrated, there is no consensus on the specific taxa most susceptible to them. Rondanelli et al. compared the effects of a very low-calorie diet (VLCD), a very low-calorie ketogenic diet (VLCKD), and a very low-carbohydrate diet (VLCarbD) on the composition of the gut microbiota. In obese individuals, disturbances in the Bacteroidetes/Firmicutes ratio were observed, leading to reduced production of short-chain fatty acids (SCFA). The use of VLCKD was associated with a decrease in the abundance of Eubacterium rectale and Roseburia and an increase in *Akkermansia muciniphil* and *Christensenellaceae* [87]. In overweight and obese men, consumption of a ketogenic diet reduced *Bifidobacterium* and Lactobacilli and increased Fusobacteria and Escherichia [81].

### 12.3. Adequate Water Supply

Water influences the functioning of the gastrointestinal tract and the composition of the intestinal microbiota, both directly (through the microorganisms present in the water) and indirectly (through chemical composition) [89]. In Europe, the recommended daily water intake is 2.0 L for women and 2.5 L for men [90]. The three main sources of water acquisition include drinking, eating, and metabolism with 80% of this coming from fluids [91]. Studies in mice by Sato K et al. have shown that limiting water intake increases intestinal transit time and alters the composition of the microbiota, increasing the abundance of bacteria from the Prevotellaceae and Verrucomicrobiaceae families (especially Akkermansia), among others [91]. In Nicaraguan children, higher contamination of drinking water with E. coli was associated with lower diversity of the gut microbiota, and lower water intake with higher presence of *Campylobacter* spp. [89]. The source of water influences the gut microbiota—people drinking well water had higher α-diversity than those drinking municipal, bottled or filtered water, while differences in β-diversity were observed both between water sources and between people drinking more or less water [90]. There were also differences in bacterial composition, with those drinking bottled or city water having more Bacteroides, Streptococcus, and Veillonella, and those drinking well water having more Dorea; those drinking less water had more Campylobacter detected [90].

## 13. Impact of Physical Activity

The gut microbiota and exercise have been shown to be linked. Endurance exercise has been shown to have positive effects on human health and on the gut microbial ecosystem, provided that the intensity of exercise is controlled [92].

The effects of exercise on individual taxa are variable but usually reveal an increase in commensal taxa such as *Bifidobacterium*, Lactobacilli, and Akkermansia [81].

Aerobic (cardio) exercise induced rapid changes in the composition of the gut microbiome, whereas resistance exercise had no effect [81].

A systematic review by Bonomini-Gnutzmann et al. (2022) [93] showed that physical activity can significantly affect the gut microbiota. Regular, moderate exercise resulted in increased microbial diversity and an increase in beneficial taxa such as *Bifidobacterium*, *Faecalibacterium prausnitzii*, *Akkermansia muciniphila*, and *Roseburia*. These changes may favor improved intestinal function and metabolism. In contrast, intense and prolonged exercise has sometimes been associated with dysbiosis and increased permeability of the intestinal barrier. Although not all studies have directly analyzed weight changes, the observed changes in the microbiota may indirectly influence weight control and metabolic parameters [93].

## 14. Diet and Long-Term Weight Loss

More and more studies indicate that diet not only affects short-term weight loss but can also lead to long-term changes in the composition of the gut microbiota, which supports lasting weight reduction effects. In the literature, weight loss up to 6 months is considered a short-term effect, while a period of more than 6 months is defined as long-term [67]. In the first phase of weight reduction, weight is reduced mainly in response to calorie restriction and modification of macronutrient composition, but data show that further weight loss or maintenance in the later period depends to a much greater extent on metabolic and molecular factors and the characteristics of the host itself, including the composition of the gut microbiota [94,95,96].

Importantly, changes in the microbiota occurring under the influence of dietary and lifestyle interventions tend to partially reverse over time after the changes are discontinued, but some beneficial modifications, such as an increase in the abundance of *Akkermansia*, may persist [97]. The initial presence of certain groups of bacteria (such as *Akkermansia*, *Alistipes*, *Symbiobacterium*, and *Pseudoflavonifractor*) may predict the success of long-term weight loss [97]. Participants who maintained their weight loss over the long term had a different microbiota profile at the outset than those who experienced the yo-yo effect, and higher abundance of bacteria such as *Alistipes* and *Symbiobacterium* and higher microbial diversity correlated with better outcomes [97,98]. The plasticity of the microbiota, i.e., its ability to adapt, also proved to be crucial—people with greater microbiological plasticity achieved better reduction results in response to a specific diet, regardless of whether it was low-fat or low-carbohydrate [94,96].

In addition, a microbiological analysis of individuals who achieved greater weight loss in the long term showed a higher presence of Bacteroidaceae *B. caccae* and *Lachnospiraceae Roseburia* NA. These are fiber-degrading bacteria whose metabolic products may help protect against weight gain and insulin resistance [67]. In contrast, individuals with better long-term outcomes showed an enrichment in genera such as *Bacteroides*, *Roseburia*, *Faecalibacterium*, and *Clostridium* XIVa, and a lower abundance of *Coriobacteriaceae*, *Streptococcus*, *Clostridiales*, *Eubacterium*, and *Coprococcus* [95].

Dietary supplementation with ingredients that affect the microbiota, such as resistant starch (RS), may further support weight loss and improve metabolic parameters, including by regulating inflammation and SCFA production, which affect energy metabolism and satiety [95,99].

In summary, the most important factors determining long-term weight loss maintenance appear to be the following: individual characteristics of the gut microbiota, its ability to adapt, and a comprehensive therapeutic approach that combines dietary changes with physical activity, behavioral support, and personalized interventions based on the initial microbiological profile. Consistent maintenance of healthy habits can significantly increase the chance of lasting therapeutic success [94,95,96,97,98,99]. These effects are summarized in Table 6.

## 15. Conclusions

The intestinal microbiota plays an important role in the regulation of metabolic, immunological and hormonal processes, and its disorders are increasingly linked to the development of obesity. Among other things, an increased ratio of Firmicutes to Bacteroidetes and reduced genus diversity of the microbiota are observed in overweight individuals. Although there is ample evidence suggesting a link between dysbiosis and obesity, the direction of this relationship is not clear—it is possible that obesity also affects the composition of the microbiota.

Promising therapeutic strategies appear to be interventions that modulate the microbiota, such as the use of probiotics, prebiotics, dietary change, physical activity or faecal microbiota transplantation (FMT). However, further clinical trials are needed to assess their efficacy and safety. A deeper understanding of the interaction between the microbiota and metabolism may contribute to the development of more effective, personalized treatments for obesity.

## Figures and Tables

**Table 1:** Factors influencing the composition of the microbiota including the predominant bacteria group and the impact on health and development [1,2,3,18,19,20,21].

Life Stage	Dominant Microbial Groups	Characteristics
👶 Newborn	*Enterobacteriaceae*, other aerobic bacteria	Low diversity, predominance of aerobic bacteria
👦 Child (~3 yrs)	Increasing numbers of *Bacteroides*, *Firmicutes*, *Bifidobacterium*	Gradual transition to adult-like microbiota
🧍‍♀️ Adult	*Firmicutes*, *Bacteroidetes* (~90%), *Actinobacteria*, *Proteobacteria*	High diversity, stable composition
👵 Older Adults	Decreased *Bifidobacterium*, reduced diversity	Higher risk of dysbiosis, impaired immune function
**Diet**		**Effect on Microbiota**
🥦 High-fiber diet, vegetables, fermented foods		Increase in *Lactobacillus*, *Bifidobacterium*
🍔 Western diet (saturated fats, sugar)		Decreased diversity, dysbiosis, predominance of potentially pathogenic bacteria
💊 Antibiotic therapy, stress, low physical activity		Disruption of microbiota balance, increased risk of metabolic diseases

**Table 2:** Systematized biochemical role played by short-chain fatty acids derived from the metabolism of the human body’s gut microbiota [28,29,30,31,32].

Aspect	Description	References/Examples
Main SCFA of interest	Butyrate	Major energy source for colonocytes
Role in intestinal barrier	Strengthens barrier integrity by upregulating tight junction proteins and stimulating mucin secretion; reduces intestinal permeability	Prevents pathogen/toxin translocation
Anti-inflammatory effects	Inhibits NF-κB activation; decreases pro-inflammatory cytokines (TNF-α, IL-6); promotes regulatory T-cell differentiation	Modulates immune responses via GPR43, GPR109a, FFAR3
Role in gut–brain axis	Influences microglial activity, neurotransmission; reduces stress and anxiety-like behavior	Supports neuroimmune regulation
Changes in disease states	Reduced butyrate production and decreased butyrate-producing bacteria (e.g., *Faecalibacterium prausnitzii*, *Roseburia* spp.) in obesity, T2D, IBD	Associated with inflammation and metabolic dysregulation
Therapeutic strategies	High-fiber diets, probiotics, fecal microbiota transplantation	Aim to increase butyrate levels and restore gut homeostasis

**Table 3:** The impact of a patient’s clinical condition on the composition of the gut microbiota and the response of the immune system [36,37,38,39].

Disease Condition	Microbiota Changes	Inflammatory/Clinical Consequences
Obesity, Metabolic Syndrome	↓ *Lactobacillus*, *Bifidobacterium*; ↑ LPS-producing bacteria	Insulin resistance, chronic inflammation, metabolic disturbances
Non-Alcoholic Fatty Liver Disease (NAFLD)	↓ Microbial diversity; ↓ *Lactobacillus*, *Bifidobacterium*; ↑ *Ruminococcus*, *Escherichia*	Increased hepatic inflammation and fibrosis progression
Colorectal Cancer (CRC)	↑ *Fusobacterium nucleatum*	Local inflammation, tumor progression, poor therapeutic response
Pediatric Leukemia (post chemotherapy/HSCT)	↓ *Bacteroides*, *Ruminococcaceae*, butyrate-producing bacteria	Gut barrier dysfunction, increased risk of GvHD and infections

**Table 4:** Differences in the composition and diversity of the gut microbiota between lean and obese individuals [26,44,48,51,55].

Feature/Bacterial Group	Lean Individuals	Obese/Overweight Individuals
Microbiota diversity	High	Reduced
*Firmicutes*/*Bacteroidetes* ratio	Lower or balanced	Often increased (though data are inconsistent)
Firmicutes	Moderate presence	Often increased
*Bacteroidetes*	Moderate to high presence	Often reduced (but not in all studies)
*Faecalibacterium*	Higher level (e.g., *F. prausnitzii*)	Reduced level
*Akkermansia*	Higher level (*A. muciniphila*)	Reduced level
*Alistipes*	Higher level	Reduced level
*Oscillibacter*	Higher level	Reduced level
*Lactobacillus reuteri*	Low level	Increased level, associated with obesity
*Lactobacillus casei*/*plantarum*	Associated with weight loss	Less common
*Prevotella*	Population-dependent (West—obesity, East—leanness)	Often higher in Western populations
*Ruminococcus*	Context-dependent	Often associated with obesity in Western countries
*Methanobrevibacter smithii (Archaea)*	More common, associated with normal weight	Less prevalent
*Bifidobacterium*	Linked to leanness (especially in Eastern populations)	Less common
*Roseburia*	Linked to leanness (in Eastern populations)	Less common
*Staphylococcus* spp.	Low presence	Increased levels, correlated with higher energy intake and CRP
Bacteroides	Moderate presence	No consistent differences

**Table 5:** Differences between the effects of probiotics, prebiotics, and synbiotics on the human body through the effect exerted by the microbiota [61,62,63,64,65,66].

Type	What Is It?	Examples	Main Benefits
**Probiotics**	Live beneficial bacteria	*Lactobacillus*, *Bifidobacterium*	Boost immunity, lower cholesterol, help with obesity and diabetes
**Prebiotics**	Nutrients that feed beneficial bacteria	Inulin, fructooligosaccharides	Promote growth of good bacteria, support weight control and gut health
**Synbiotics**	Combination of probiotics and prebiotics	Combinations of both	Synergistic effects, support weight loss and metabolic health

**Table 6:** Overview of selected therapeutic methods and nutrients on changes in body weight and clinical condition of the patient.

Intervention	Description/Application	Effects	Evidence Strength
**Probiotics**	Supplementation with strains such as *Lactobacillus*, *Bifidobacterium*, Enterococcus	Reduction in BMI, body weight, cholesterol, immune support; effect weaker than drugs, but fewer side-effects	Confirmed in numerous clinical studies, moderate effectiveness [67]
**Prebiotics**	Dietary components (e.g., inulin, FOS) stimulating the growth of beneficial bacteria	Reduction in body and fat mass (especially in animal studies); confirmed effect on microbiota	Weaker effect than probiotics, confirmed [66,67]
**Synbiotics**	Combination of probiotics and prebiotics	Supporting reduction of body weight and BMI	Confirmed effectiveness, moderate effect [67]
**FMT (Fecal Microbiota Transplantation)**	Microbiota transplantation from a lean donor to an obese person	In animals: improvement of metabolic parameters; in humans: change in microbiota, BUT: no effect on BMI/metabolic parameters in the short term	Well-documented efficacy for C. difficile; limited and inconclusive for obesity and metabolic diseases [55,72,74]
**High-fiber diet**	Increased fiber intake (plants, whole grains)	Increased microbiota diversity, lower long-term risk of weight gain	Many studies confirm positive effects on microbiota and weight [83]
**Varied protein sources**	More plant, less animal protein	Increase in beneficial bacteria, SCFA production	Confirmed microbiota changes, less certain effect on weight [83]
Fats	More unsaturated, less saturated fat	Unsaturated: increase in beneficial bacteria; saturated: dysbiosis	Effects on microbiota documented, impact on weight depends on fat type [19,80]
Ketogenic diet	Very low carbohydrate, high fat	Changes in microbiota, inconclusive results, effect on weight documented	Effect on microbiota and weight: inconclusive, mixed results [81,86,87,88]
Proper hydration	Amount and source of drinking water	Impact on microbiota diversity, pathogenic bacteria	Studies in humans and animals—confirmed effect [89,90,91]
Physical activity	Regular, moderate exercise	Increased diversity and beneficial bacteria, positive effect on metabolism	Confirmed positive effect on microbiota [92,93]
